# Human papillomavirus (HPV) type 16 E7 protein bodies cause tumour regression in mice

**DOI:** 10.1186/1471-2407-14-367

**Published:** 2014-05-24

**Authors:** Mark Whitehead, Peter Öhlschläger, Fahad N Almajhdi, Leonor Alloza, Pablo Marzábal, Ann E Meyers, Inga I Hitzeroth, Edward P Rybicki

**Affiliations:** 1 Department of Molecular and Cell Biology, University of Cape Town, Private Bag X3, Cape Town, Rondebosch 7700, South Africa; 2 Department of Chemistry and Biotechnology, Aachen University of Applied Sciences, Jülich 52428, Germany; 3 Department of Immunology, University of Konstanz, Constance, Germany; 4 Molecular Virology Department, Botany and Microbiology College of Science, King Saud University, Riyadh 11451, Saudi Arabia; 5 ERA Biotech, Parc de Recerca UAB Bellaterra, Barcelona, Spain; 6 Institute of Infectious Diseases and Molecular Medicine, University of Cape Town, Rondebosch 7700, South Africa

**Keywords:** Cervical cancer, DNA vaccine, HPV-16, E7, Zera® protein, Protein body, Plant-produced

## Abstract

**Background:**

Human papillomaviruses (HPV) are the causative agents of cervical cancer in women, which results in over 250 000 deaths per year. Presently there are two prophylactic vaccines on the market, protecting against the two most common high-risk HPV types 16 and 18. These vaccines remain very expensive and are not generally affordable in developing countries where they are needed most. Additionally, there remains a need to treat women that are already infected with HPV, and who have high-grade lesions or cervical cancer.

**Methods:**

In this paper, we characterize the immunogenicity of a therapeutic vaccine that targets the E7 protein of the most prevalent high-risk HPV - type 16 – the gene which has previously been shown to be effective in DNA vaccine trials in mice. The synthetic shuffled HPV-16 E7 (16E7SH) has lost its transforming properties but retains all naturally-occurring CTL epitopes. This was genetically fused to Zera®, a self-assembly domain of the maize γ-zein able to induce the accumulation of recombinant proteins into protein bodies (PBs), within the endoplasmic reticulum in a number of expression systems.

**Results:**

High-level expression of the HPV 16E7SH protein fused to Zera® in plants was achieved, and the protein bodies could be easily and cost-effectively purified. Immune responses comparable to the 16E7SH DNA vaccine were demonstrated in the murine model, with the protein vaccine successfully inducing a specific humoral as well as cell mediated immune response, and mediating tumour regression.

**Conclusions:**

The fusion of 16E7SH to the Zera® peptide was found to enhance the immune responses, presumably by means of a more efficient antigen presentation via the protein bodies. Interestingly, simply mixing the free PBs and 16E7SH also enhanced immune responses, indicating an adjuvant activity for the Zera® PBs.

## Background

Cervical cancer is the second most important cause of cancer-related deaths in women, with half a million diagnosed cases and more than 250 000 deaths recorded each year. This is a direct result of Human papillomavirus (HPV) infections of the cervical epithelium. The high risk HPV types 16 and 18 are most prevalent globally in cervical infections, and are linked to more than 50% and 20% of all cervical cancers, respectively [1]. There are currently two licensed prophylactic vaccines available: Cervarix (GlaxoSmithKline) protects against high-risk types HPV-16 and 18 only, while Gardasil (Merck) protects against HPV-16 and 18, as well as the HPV types 6 and 11 that are the most common viruses associated with genital warts. These vaccines have been shown to be very effective in preventing the onset of cervical cancer, and they are well tolerated [2]. However, they are limited in that they only protect against the two most prevalent high risk HPV types: there are over a hundred different HPV types, of which about 40 are known to infect the genital tract and 12 have been linked to cervical cancer [3]. These vaccines are also not particularly suitable for dissemination in developing countries, primarily due to their high cost to individuals or to state vaccine schemes. Additionally, both of these prophylactic vaccines only prevent infection, and are not therapeutic for those already infected. Therefore, there is still an urgent need for low-cost and particularly for therapeutic HPV vaccines.

The role of therapeutic vaccines against HPV is to promote regression of HPV-related lesions. They should therefore elicit cell-mediated immune responses that are capable of recognizing HPV-infected and transformed epithelial cells, as opposed to the antibody-based humoral responses elicited by prophylactic vaccines. Preferentially, the vaccines should elicit cytotoxic T-lymphocyte (CTL) responses which act to eradicate infected cells [4]. However, as antibodies can also be involved in the eradication of infected cells by antibody-dependent cytotoxicity (ADCC), an optimal therapeutic vaccine should induce both a humoral as well as a cellular immune response [5,6].

The HPV E7 oncoprotein has become a primary focus as a therapeutic vaccine target, due to its constitutive and exclusive expression by HPV-infected cells generally, and in particular by cervical cancers and the premalignant dysplasic cells [7]. E7 is a nuclear protein of 97 amino acids in size, and plays a role in inducing DNA synthesis in cells partly by means of binding hypophosphorylated retinoblastoma protein (pRB). This disrupts the interaction between elongation factor 2 and the pRB, causing the cell to shift to the S phase of the cell cycle [8]. In cervical cancers and high-grade lesions the HPV genome is very often integrated into the host genome, leading to inactivation of the early gene E2 (responsible for regulating transcription) and subsequent activation of the oncogenic E7 gene. The E7 protein is then persistently expressed and causes the immortalization of primary keratinocytes, leading to terminally differentiated, immortalized clones [9].

In the past, DNA vaccines have clearly demonstrated they have the ability to induce remarkable CTL responses [10], and are thus thought to be interesting candidates for therapeutic vaccines. Öhlschläger et al. [11] have shown that immunization of mice with a “shuffled” HPV-16 E7 gene did not cause cell proliferation, but induced strong HPV-16 E7-wildtype-specific cellular and humoral responses without the use of any adjuvant.

Although these findings demonstrated the potential of therapeutic HPV DNA vaccines, there has been limited success observed in clinical trials so far [12-14]. Moreover, there are concerns about the potential integration of the injected DNA into the host genome leading to long-term complications. Various protein-based HPV therapeutic vaccines have moved to clinical trials [12,14]; however, there remain concerns over the cost of cell culture-produced proteins in the context of developing country needs.

It is thought that plant-produced proteins could provide an alternative to DNA-based vaccines because they are cheap, effective [15,16] and, moreover, have been proven safe for use in humans [17]. A proof of efficacy for a prophylactic plant-produced papillomavirus L1 protein vaccine was provided by Kohl et al. [18], who showed protection in New Zealand White rabbits against warts caused by cottontail rabbit papillomavirus (CRPV) [18]. HPV 16 L1 protein that assembles into highly immunogenic virus-like particles (VLPs) and elicits neutralising antibodies has been produced successfully at high yield via transient expression in *Nicotiana benthamiana*[19]. A number of groups have also investigated the plant production of E7-based therapeutic vaccines against HPV-16, with significant success in murine tumour models ([20,21]. In particular, a plant viral vector/*N. benthamiana*-produced E7 mutant (E7GGG, cannot bind Rb) fused to the *Clostridium thermocellum* β-1,3-1,4-glucanase (LicKM) expressed at high yield, elicited E7-specific humoral and CTL responses in mice, and was both protective against E7-expressing tumour cell challenge, and therapeutic against existing tumours [20].

Transient expression systems for recombinant proteins in whole plants are useful because they allow high-level production of protein in just a few days, and are easily scalable [16,22]. Methods for increasing protein yield include codon optimisation of the genes, and fusion of signal sequences to target recombinant proteins to subcellular compartments [18,19]. Signal sequences fused with the gene of interest can increase protein accumulation and provide protection from degradation by host cell enzymes.

Additionally, certain sequences may drive assembly and subsequent sequestration of the polypeptide into large and highly protected “protein bodies”. One of the storage proteins of the maize kernel, γ-zein, is naturally accumulated at high levels into the endoplasmic reticulum (ER). The functional domains of γ-zein have been well described [23]. The N-terminal domain, containing eight PPPVHL repeats and a Pro-X sequence, allows ER retention and accumulation of fusion proteins in membrane-defined protein bodies, and may also determine interaction with membranes. The C-terminal cysteine-rich domain has been hypothesized to have a role in the final “packing” of the protein bodies due to the formation of inter- and intra-chain disulfide bonds. The Zera® sequence generated from the maize γ-zein sequence has been described as being sufficient to induce retention of recombinant proteins in protein bodies called StorPro® organelles (ERA Biotech, Spain), allowing better accumulation of fusion proteins. In addition, the formation of the large, stable protein body makes it considerably simpler to concentrate and purify the protein of interest [24,25]. A further advantage of such protein bodies is that their co-administration with recombinant vaccines may have an adjuvant effect and enhance the immune response as a result of their particulate nature.

We explored the development of a plant-produced, potentially therapeutic protein-based vaccine that could cause regression of HPV lesions in humans infected with HPV-16, and which would also be affordable in developing countries. We investigated whether HPV-16E7SH and Zera® protein bodies can induce tumour regression in mice. This was tested either by administering 16E7SH-Zera® fusion proteins or by administering a mixture of Zera® protein bodies (PBs) and 16E7SH protein to tumourigenic mice. The proteins were produced in three different expression systems: HPV-16E7SH protein fused to Zera® was expressed in plants, HPV-16E7SH was produced in *E. coli* and Zera® PBs were produced in insect cells. Immune responses of the plant-produced protein were compared to those of the well-characterised E7SH DNA vaccine in the murine model; tumour regression as well as cell-mediated and humoral responses were analysed. Finally, the adjuvanting properties of the Zera® protein were investigated.

## Methods

### Plasmid construction

**Figure 1:**
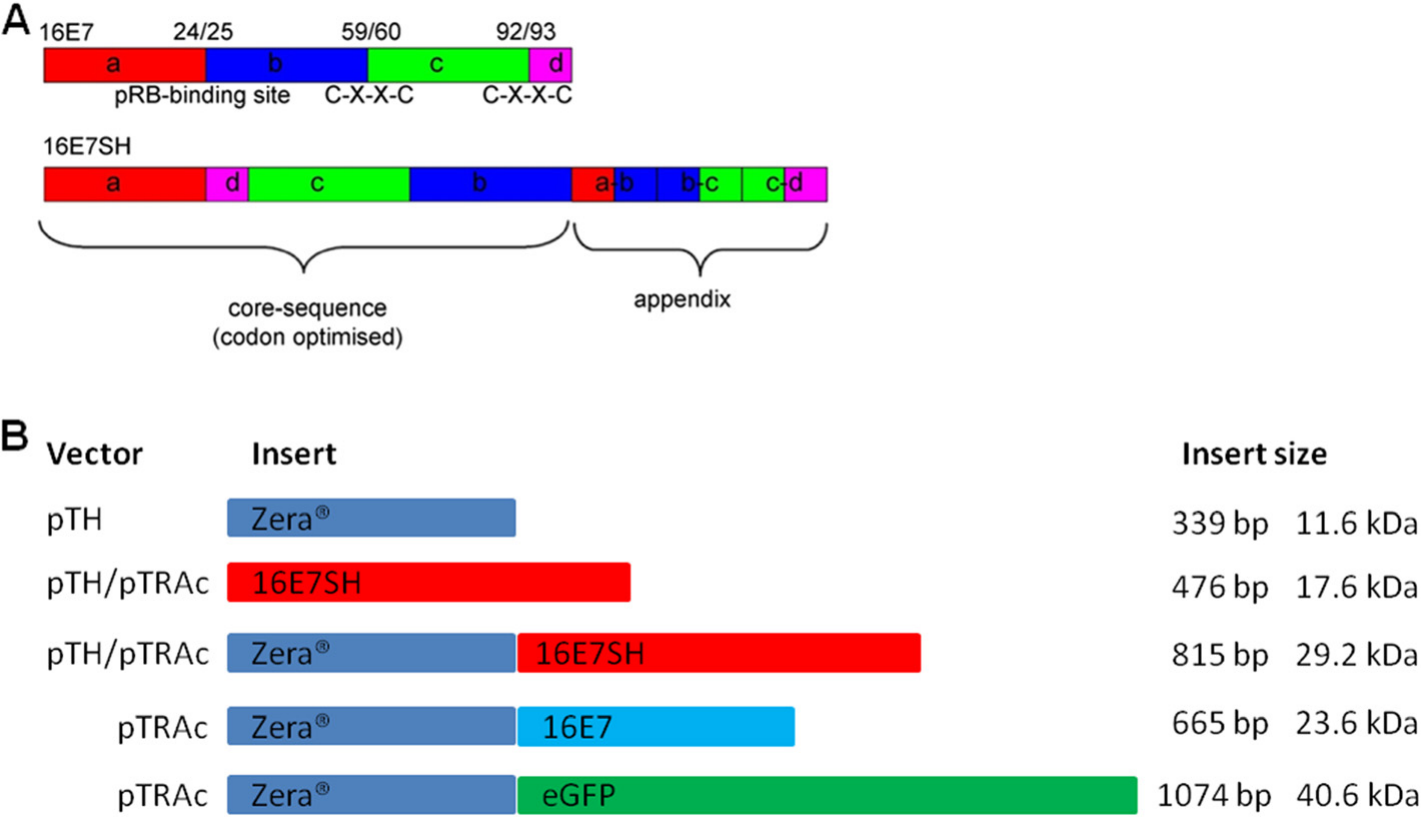
**Constructs used for making DNA vaccines. (A)** The HPV-16 E7 gene was cleaved at the positions corresponding to the pRB binding site and in between the two Cys-XX-Cys motifs. The cleavage points between amino-acid numbers are shown above the gene. The resulting fragments were rearranged (“shuffled”) forming the core-element, and an appendix containing the junctions where the cleavage took place was added to avoid loss of putative CTL epitopes to form 16E7SH. **(B)** Depiction of the 7 constructs utilised in this study. The core genes and fusions of genes were cloned into the respective pTH or pTRAc expression vectors.

The construct into which the 16E7 and 16E7SH genes were cloned for expression in plants (pTRAc-ZERA-eGFP) was made as follows: the eGFP gene was amplified from pEGFP (BD Biosciences) using a forward primer (5’-gatcccatg*gacgacgatgataag*gtgagcaagggcgaggagctg-3’) which allowed for inclusion of an enterokinase cleavage site (DDDDK) at the 5’ terminus of eGFP (italicized sequence), and the following reverse primer: 5’ cggatccattacttgtacagctcgtccatgccgag 3’. The amplified product was subcloned into pUC18ZERA (provided by Era Biotech, Barcelona, Spain [26] using *Nco* I and *Bam*H I restriction enzyme sites such that the Zera® sequence was in frame with the eGFP fusion to generate pZERA-GFP. This construct was amplified using primers (5’ actcatgagggtgttgctcgttgc 3’ and 5’ cggaccattacttgtacagct 3’) to enable cloning of the ZERA-eGFP fusion into the plant expression vector pTRAc (provided by Rainer Fischer, Fraunhofer Institute, Molecular Biology and Applied Ecology, Aachen, Germany) [19], using *Bsp*H I and *Bam*H I restriction enzyme sites. The oncogenic HPV-16 E7 (*16E7*) gene (Figure 1A) was provided by J. Schiller (Laboratory of Cellular Oncology, National Cancer Institute, Bethesda, MD, USA) and amplified using primers 5'-gatc*tcatgagtgacgacgatgataag*atgcatggagatacacctacattg-3'and 5'-ag*ggatcc*ttatggtttctgagaacagatgg-3'. The product was cloned into the *Nco* I and *Bam*H I sites of pTRAc-ZERA-eGFP (replacing eGFP) forming pTRAc-ZERA-16E7. The shuffled HPV 16E7 (*16E7SH*) gene (Figure 1A) [11] was amplified from pTH-16E7SH using the following primers: 5'-gatc*ccatgg*acgacgatgataagatgcacggcgacaccccc-3' and 5'- aa*ggatcc*ttatggtttctgagaacagatggggcac-3'. The product was cloned into the *Nco* I and *Bam*H I sites of pTRAc-ZERA-eGFP (replacing eGFP) forming pTRAc-ZERA-16E7SH. In addition, the modified 16E7SH fragment was cloned into the *Afl* III and *Bam*H I sites of the vector pTRAc, yielding pTRAc-16E7SH.

To construct the recombinant DNA vaccine, *ZERA-16E7SH* was amplified from pTRAc-ZERA-16E7SH with primers 5'-aa*aagctt*catgagggtgttgctcgttg-3' and 5'-at*gaattc*t*ggatcc*ttatggtttctgag-3' and cloned into the pTH vector [27] using *Hin*d III and *Eco*R I to yield pTH-ZERA-16E7SH. The constructs utilised in this study are summarised in Figure 1B. For expression in *E. coli*, 16E7SH was cloned into pPROEX™ HT (Life Technologies) and for expression in insect cells it was cloned into flashBAC expression vector (Oxford Expression Technologies Ltd.).

### *Agrobacterium* transformation

Three hundred ng of the pTRA constructs were individually electroporated into *A. tumefaciens* GV3101 strain with the pMP90RK helper plasmid as previously described [19]. Electroporated cells were incubated in 1 ml Luria–Bertani (LB) broth for 2 h then plated on LB medium containing, 50 μg rifampicin ml^-1^ and 30 μg kanamycin ml^-1^.

### Agroinfiltration and transient expression

*Agrobacterium* LBA4404 with pBIN-NSs, containing the TSWV NSs silencing suppressor gene [28], and *Agrobacterium tumefaciens* GV3101::pMP90RK with the pTRAc constructs were grown in induction medium, prepared for infiltration and the *Agrobacterium* suspension was either injection- (small scale - a few leaves) or vacuum-infiltrated (large scale - whole plants) into the abaxial air spaces of 6-8 week old *N. benthamiana* leaves and left to grow under 16 h light, 8 h dark at 22°C growth conditions all previously described until the desired extraction time ranging from day 1 to day 10 post infiltration [19]. The constructs were either infiltrated alone or co-infiltrated with the LBA4404 pBIN-NSs.

### Protein extraction

To screen leaf tissue for protein expression, five leaf discs (5 mm diameter ~ 0.05 g wet plant mass) were ground in liquid nitrogen, incubated in 200 μl of extraction buffer (100 mM Tris pH 8, 5% SDS, 5% β-ME, 200 mM NaCl) with 1× Complete Protease Inhibitor (EDTA-free; Roche) at 95°C for 20 min. Samples were then incubated at RT for 1 h followed by agitated incubation at 37°C overnight. The supernatant was clarified by centrifugation for 20 min (13000 rpm, desktop centrifuge, 4°C) and then detected by means of western blots.

### Protein detection in leaf extracts

Samples were incubated at 85°C for 5 min in loading buffer, separated on 15% SDS-PAGE, then either stained with Coomassie blue or transferred onto a nitrocellulose membrane using the Trans-Blot® SD Semi-Dry Transfer Cell (Bio-rad) for western blot analysis. E7 proteins were detected with anti-E7 sera (1:4000) followed by goat anti-mouse-alkaline-phosphatase conjugate (1:10000; Sigma). Zera®-containing proteins were detected with polyclonal anti-Zera® sera (1:5000; ERA biotech) followed by goat anti-rabbit-alkaline-phosphatase conjugate (1:5000; Sigma). NBT/NCIP tablets (Roche) were used for final detection.

Proteins were quantified by measuring the density of the band on a western blot or Coomassie stained bands in comparison to a known protein concentration standard, using GeneTools software (SYNGENE) on scanned images. TSP was determined using the BioRAD assay according to the manufacturer's instructions.

### Large-scale expression and sucrose gradient purification of protein bodies (PBs)

Large-scale expression and purification was required to produce vaccine dosages for animal trials. Seven days post vacuum infiltration, the leaves were cut, weighed, ground in liquid nitrogen using a pestle and mortar and resuspended in a ratio of 1:5 (w/v) of buffer PBP3 (100 mM Tris pH 8, 50 mM KCl, 6 mM MgCl_2_, 10 mM EDTA, 0.4 M NaCl) made up in 10% sucrose. Samples were centrifuged at 24 000 rpm for 10 min on ice and then filtered through a Miracloth™ (Calbiochem). The filtrate was loaded onto a sucrose step gradient (19%, 27%, 42%, 56% w/w) and ultracentrifuged at 80 000 g, 4°C for 2 h (Beckman SW32Ti rotor). Protein fractions (IF) of 2 ml were retrieved at the step interface and the pellet was resuspended in 2 ml PBP3 and analyzed by western blotting.

### Expression of 16E7SH in *E. coli*

16E7SH protein for animal trials was expressed in *E. coli* as detectable levels of its expression in plants were never achieved. Competent DH5α *E. coli* cells were transformed and protein expression was induced as per manufacturer’s recommendations. The induced cells were pelleted and lysed (Tris-HCl 50 mM pH 8, NaCl 300 mM,5% glycerol,1 mM DTT). Inclusion bodies containing 16E7SH were solubilized with solubilization buffer (Tris-HCl 50 mM pH 7.6, NaCl 300 mM, 8 M Urea, 2 mM DTT). After solubilization, 16E7SH was IMAC-purified twice on a Ni column, washing bound samples with Triton X-114, both to purify the protein and to remove endotoxins. Eluted proteins were further purified twice by size exclusion chromatography (Superdex 200 column, GE Healthcare Life Sciences), the first in the presence of arginine, and the second with phosphate-buffered saline (PBS). The resulting protein was analyzed to verify the absence of LPS contamination with an Endosafe®-PTS™ test system (Charles River Ltd.).

### Insect cell culture and baculovirus production of PBs

*Spodoptera frugiperda* (*Sf*9) insect cells (Invitrogen) were grown in suspension or as monolayers at 28°C in serum-free SF900 SFM Medium (Gibco). Recombinant baculoviruses were produced by co-transfection of *Sf*9 cells with flashBAC DNA and transfer vectors pBacPak8 containing the Zera®-encoding sequence, according to the manufacturer’s recommendations. Recombinant viruses were titrated and monolayer *Sf*9 cultures were infected with the recombinant baculovirus at a multiplicity of infection (MOI) of 5.

Zera® protein bodies were isolated from frozen *Sf*9 cell biomass previously infected with the selected baculovirus. Zera® PBs were recovered as described by Torrent et al. [26]. PBs washed with LPS-free water were characterized by SDS-PAGE and confocal and scanning electron microscopy.

### Mammalian cell culture

Wildtype HPV-16 E7-expressing 2 F11 cells (C57BL/6 origin, H2b haplotype; [29] were cultured in RPMI 1640 supplemented with heat-inactivated 5% (v/v) foetal calf serum (FCS, Gibco, Eggenstein, Germany), 2 mM L-glutamine, penicillin (100 U/ml) and streptomycin (100 μg/ml), G418 (0.8 mg/ml). RMA cells [30] were cultured with the same medium with the exception of G418.

C3 tumour cells derived from embryonic mouse cells transfected with the complete HPV-16 genome [31] were cultured in the same medium as 2 F11 cells, supplemented with kanamycin (0.1 mg/ml). Splenocytes were cultured in αMEM (Sigma, Deisenhofen, Germany) supplemented with 10% FCS, 0.1 mM β-mercaptoethanol, 4 mM glutamine and antibiotics as above for the first 4-5 days after splenectomy. Subsequently, the splenocytes were cultured in αMEM + supplemented with 2.5% supernatant of a concanavalin-A-induced rat spleen cell culture as a source of murine IL-2 and 25 mM methyl-α-mannopyranosid (Sigma, Deisenhofen, Germany).

### Immunization of mice

Six-to-eight week old female C57BL/6 mice (owner bred) were kept under SPF isolation conditions and standard diet at the animal facilities of the University of Konstanz, Konstanz, Germany. In the case of DNA injections, agarose-gel verified plasmids (>95% supercoiled) of preparations containing less than 0.1 endotoxin units/μg plasmid DNA as tested earlier by Limulus endotoxin assay (QIAGEN EndoFree Plasmid Kit). PBs were thoroughly sonicated on ice. For co-inoculation with PBs and E7 proteins, PBs were mixed with the recombinant homogenized E7 protein by pipetting on ice directly prior to immunization (2-4 minutes). For CTL analysis animals were immunized once (100 μg DNA/per animal [50 μg DNA in 50 μl PBS per *musculus tibialis anterior i.m.*] or 5 μg ZERA-16E7SH +/- 100 μl Incomplete Freund’s adjuvant (IFA) or 2.5 μg 16E7SH +/- 2.5 μg Zera® PBs +/- 100 μl IFA per animal s.c. into the left flank). Ten to 12 days after vaccination animals were sacrificed and spleens were isolated.

### Tumour regression experiments

C57BL/6 mice received 0.5 × 10^6^ HPV-16 E7 expressing C3 cells [31] in 100 μl of PBS, subcutaneously in the right shaved flank (needles: 20G 1½” BD Microlance 3). When small tumours were palpable in all animals (12-15 days after tumour cell injection), the vaccine was injected i.m. in both *musculus tibialis anterior* for the DNA vaccines, or s.c. into the left flank for protein vaccines, as described above. Tumour sizes were measured with a caliper. Mice were sacrificed when the tumour size reached 400 mm^2^ or when tumours were bleeding. Tumour sizes of the mice within a group were calculated as arithmetic means with standard deviation (SD). All operations on live animals were performed under Isoflurane anaesthesia.

All animal experiments were performed with approval by and in accordance with regulatory guidelines and standards set by the institutional review board at Regierungspraesidium, Freiburg, Germany.

### CTL and humoral responses in vaccinated mice

Data provided were obtained without *in vitro* restimulation *ex vivo,* all ELISPOT assays, or after one *in vitro* restimulation (^51^Cr-release assay). In the case of *in vitro* restimulation, 2 × 10^7^ splenocytes (pretreated with ACT lysis buffer [17 mM Tris/HCl, 160 mM NH_4_Cl, pH 7.2] to deplete erythrocytes) were co-cultured with 2 × 10^6^ irradiated (100 Gy) HPV-16 E7 wildtype-expressing 2 F11 cells [29] cells in 25 cm^2^ culture flasks for 5-6 days. Cultures were grown at 37°C and 7.5% CO_2_ in a humidified incubator.

### IFN-γ/Granzyme B ELISPOT assays

Murine IFN-γ ELISPOT assays were performed *ex vivo* as previously described [11]. The Granzyme B ELISPOT assay was performed similarly to the IFN-γ ELISPOT assay. For this assay, the anti-mouse Granzyme capture antibody (100 ng/well, AF1865; R&D Systems, Minneapolis, USA) and the biotinylated anti-mouse Granzyme detection antibody (50 ng/well, BAF1865; R&D Systems, Minneapolis, USA) were used. Splenocytes were seeded in triplicate in 2-fold serial dilutions from 200 000 to 25 000 cells per well. One of the triplicates was left untreated (negative control), the second received 200 ng of pokeweed mitogen/well (Sigma, Deisenhofen, Germany) in 2 μl of PBS (positive control), whereas the third received 0.2 μmol of H2Db-restricted HPV-16 E749-57 peptide in 2 μl of PBS/well (test sample). Spots of the negative control (untreated) were subtracted from the spot number in the corresponding test sample.

### ^51^Cr-release assays

The ^51^Cr-release assays were performed after one *in vitro* restimulation of murine spleen cells. One × 10^4^ Na_2_CrO_4_-labelled (0.05 mCi) target cells/well (RMA or E7 wildtype expressing 2 F11 cells) were incubated together with decreasing numbers of effector cells in 200 μl per well of a 96-well round bottom plate (Costar, Corning, USA) for 4 h. Subsequently, 50 μl of supernatant was harvested from each well and the released radioactivity was measured in a Microbeta counter (Wallac, Turku, Finland). Specific lysis was calculated according to the formula: percent specific lysis = [(cpm of the sample - spontaneous release)/(total release - spontaneous release)] × 100, where total release and spontaneous release are measured in counts per minute (cpm). Spontaneous chromium release was determined by using ^51^Cr-labeled target cells without effector cells, and total chromium release was determined by adding 2% Triton X-100 to lyse the labelled target cells.

### Humoral antibody titre determination by ELISA

One μg/ml of recombinant HPV-16 E7-wildtype protein (ProteinX Lab, San Diego, CA, USA, Cat. No. 2003207) or Zera® protein diluted in PBS was used to coat round-bottom enzyme-linked immunosorbent assay (ELISA) plates (Becton Dickinson) by incubating at 4°C overnight. Wells containing PBS were used as a negative control. Plates were washed three times with PBS containing 0.05% Tween 20 and incubated for 1 h at 37°C with 100 μl of milk buffer (5% milk powder and 0.05% Tween 20 in PBS) per well. After the wells were washed three times with PBS, serum specimens diluted 1:10 in milk buffer, post-immunization [day of splenectomy] were added to two wells in a total volume of 50 μl per well, and incubated for 1 h at 37°C. Samples were removed and washed three times with PBS. In order to detect IgGs, HRP-conjugated rabbit anti-mouse IgG (heavy and light chains) (Zymed, San Francisco, Calif.) diluted 1:3000 were used. After incubation for 1 h at 37°C and three washes with PBS, substrate (200 μg of tetramethylbenzidine per ml in a solution of 0.1 M Na acetate [pH 6.0] and 0.03% H_2_O_2_) was added, the reaction was stopped with 1 M H_2_SO_4_, and the plates were assayed in an ELISA reader at 450 nm.

### Statistical analysis

Differences of means between experimental and control group were considered statistically significant when p was less than 0.05 by unpaired Students t-test.

## Results

### Expression and accumulation of plant-produced recombinant proteins

In order to determine the protein expression profiles of the recombinant HPV constructs over time, and the extent of protection that the PB structures provide for the recombinant protein against host proteolytic degradation, protein expression levels in crude leaf extracts were compared by western blotting.

**Figure 2:**
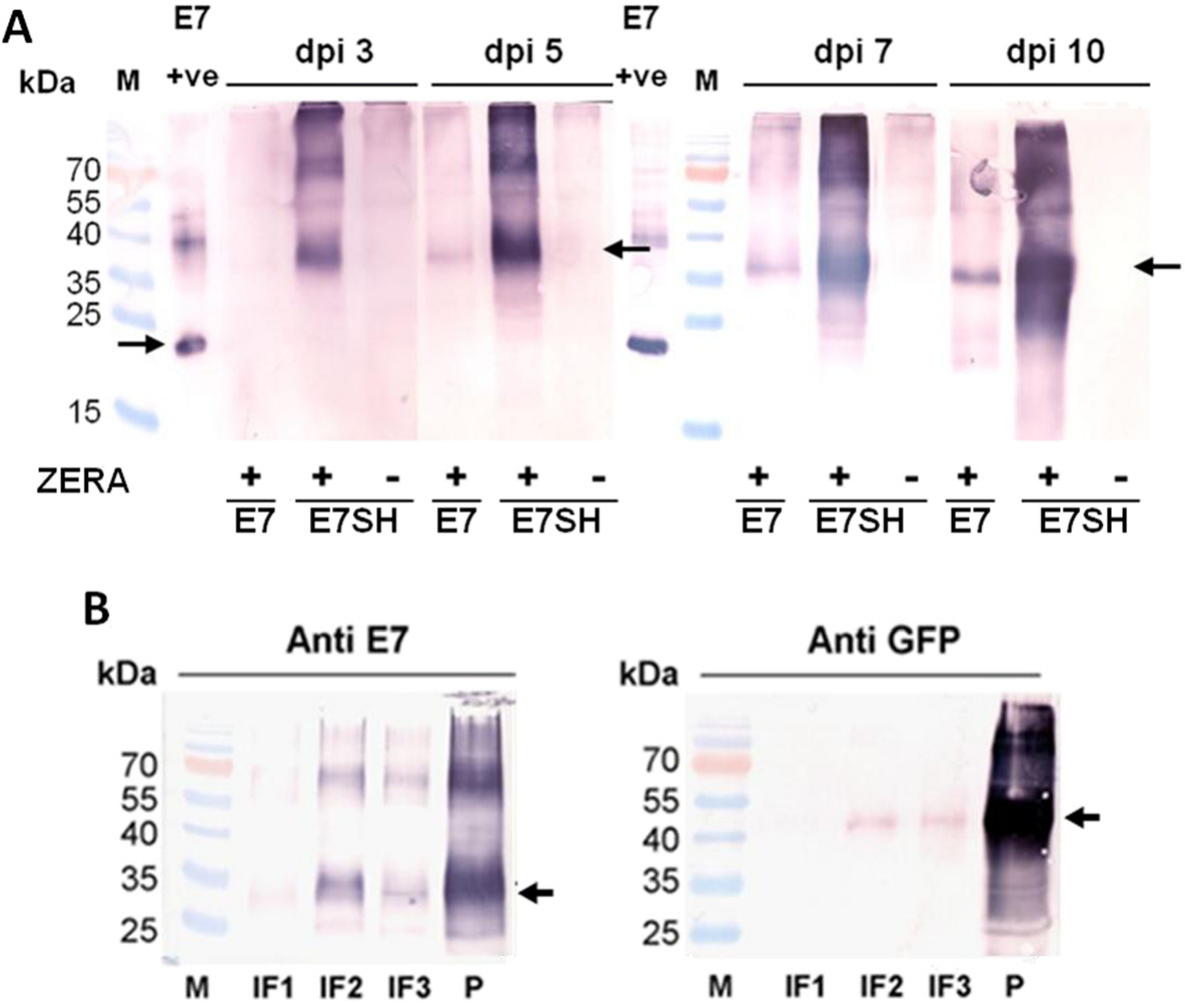
**Western blots of crude plant extracts and of purified ZERA-16E7SH and ZERA-eGFP protein. (A)** Samples from plants expressing ZERA-16E7, ZERA-16E7SH and 16E7SH were harvested at 3, 5, 7 and 10 dpi, separated on a polyacrylamide gel and blotted onto nitrocellulose membrane. Proteins were detected with HPV-16 E7 antibody. 0.6 μg of *E. coli-* produced purified His-E7 protein was used as a positive (+ve) and comparative control. SH indicates the use of the shuffled 16E7 gene. + or - indicates the fusion of Zera®. Black arrows indicate the E7 positive control protein (18 kDa) and the Zera®-fused E7 proteins. **(B)** Leaves from vacuum-infiltrated *N. benthamiana* co-infiltrated with pBIN-NSS and either pTRAc-ZERA-16E7SH or pTRAc-ZERA-eGFP were extracted 5 dpi, ground in liquid nitrogen, homogenized, filtered and separated on sucrose density gradients. Interphase fractions (IF) were aspirated, pellets (P) were resuspended and run on acrylamide gels, blotted on nitrocellulose membranes and probed with HPV-16 E7 monoclonal antibody or anti-GFP monoclonal antibody. Black arrows indicate protein bands of expected sizes.

The constructs (pTRAc-16E7SH, pTRAc-ZERA-16E7SH, pTRAc-ZERA-16E7 and pTRAc-ZERA-GFP) were transiently expressed in 8-week old *N. benthamiana* plants after syringe co-infiltration of each experimental recombinant *Agrobacterium* strain with a strain expressing the silencing suppressor NSs. Proteins were extracted at 3, 5, 7 and 10 dpi. The pTRAc-16E7SH, pTRAc-ZERA-16E7SH and pTRAc-ZERA-16E7 samples were analyzed by immunoblotting with the HPV 16E7 antibody (Figure 2A). Concentrations were estimated by comparison with 0.6 μg of bacterially-expressed 16E7SH protein (≈18 kDa) used as a positive control by means of densitometry.

ZERA-16E7 protein (expected size of 23 kDa) was not detected by western blotting in samples harvested at 3 dpi. However, this protein was observed at 5 dpi, and accumulation increased gradually through 7 dpi to 10 dpi, with a maximal accumulation of 150 mg/kg measured by gel densitometry. ZERA-16E7SH protein (expected size of 29 kDa) was seen at 3 dpi and accumulated to much higher concentrations, starting with a concentration of 400 mg/kg at 3 dpi and gradually increasing to 1100 mg/kg at 10 dpi. For further purification studies and preparation of recombinant proteins for animal experiments, infiltrated plants were harvested at 7 dpi, as this was the time at which the highest protein levels were visualised.

In contrast, 16E7SH protein alone (expected size of 18 kDa) was not detected throughout the same time trials, indicating the positive effect of the Zera® peptide on the accumulation of the fusion protein (Figure 2A). A similarly-loaded gel probed with anti-Zera® antibody reacted with the appropriate Zera®-containing proteins, verifying the presence of Zera® -specific epitopes on these particular proteins (data not shown).

### Purification of protein bodies for animal trials

Plant leaves were vacuum-infiltrated with pTRAc-ZERA-16E7SH or pTRAc-ZERA-eGFP, and PBs were purified at 7 dpi. Extracts from these infiltrated plants were ultracentrifuged on a sucrose density step gradient and the interphase fractions (IF) were aspirated, and tested by western blotting. Pelleted material at the bottom of the gradient was also tested by western blotting. Blots were probed with either anti-16E7 or anti-GFP antibodies to examine where ZERA-16E7SH and ZERA-eGFP PBs were positioned on the gradient (Figure 2B). For both products, the highest levels of recombinant protein were found in the pellets (P): ZERA-16E7SH could be purified at 50 mg/kg, and ZERA-eGFP at 200 mg/kg as measured by densitometry. In both cases, low levels of recombinant protein were detected using anti-E7 antibody in fractions two (IF2) and three (IF3) and an even lower amount in IF1. Pellets containing the ZERA-16E7SH PBs were resuspended in endotoxin free PBS and used in mouse experiments.

### Tumour regression experiments in mice inoculated with plant-produced ZERA-16E7SH

**Figure 3:**
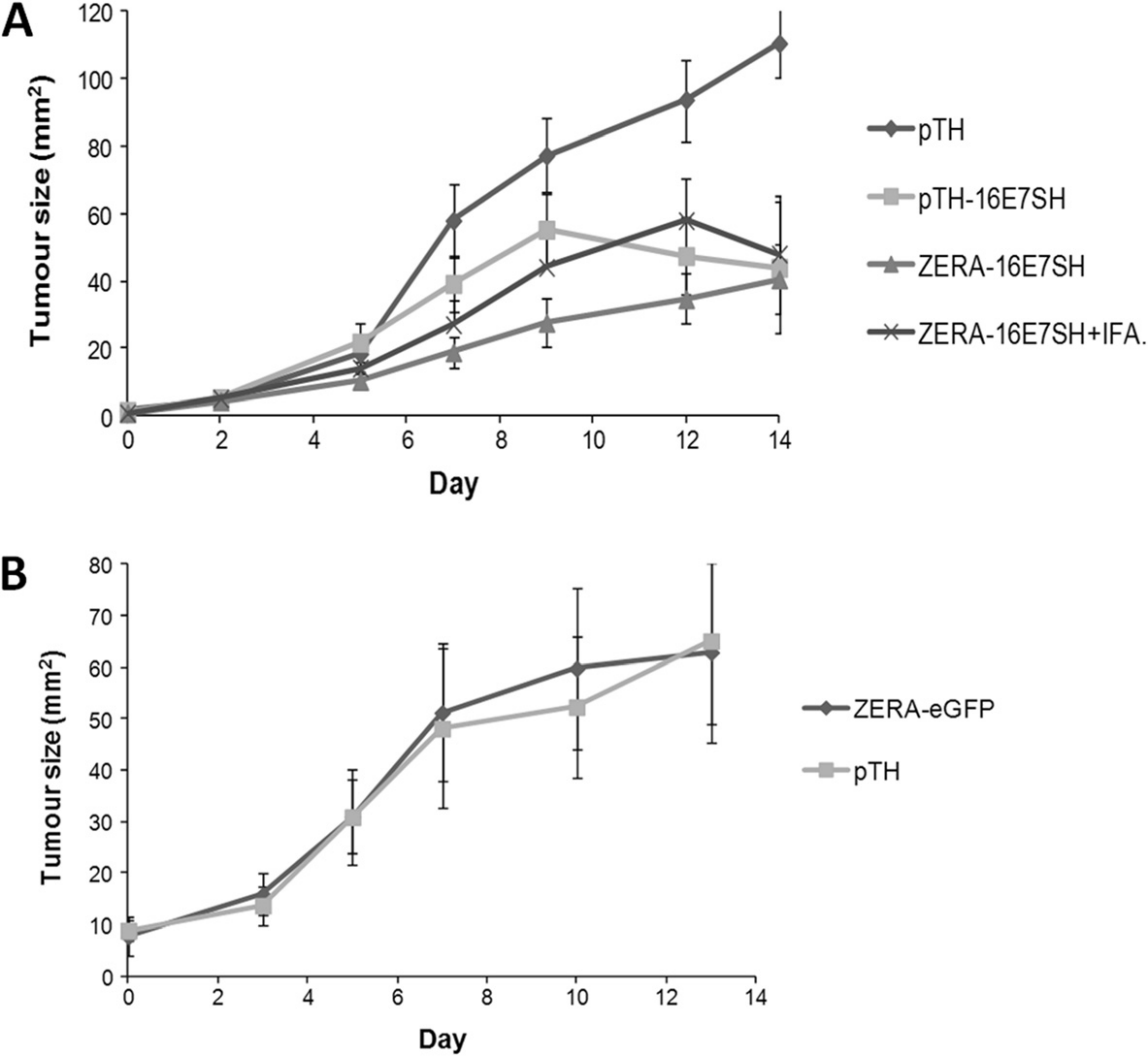
**Tumour regression in mice inoculated with different vaccines.** Mice were inoculated with 0.5 × 10^6^ C3 tumour cells to induce tumours and subsequently injected with vaccine (5 μg protein or 100 μg DNA in 100 μl) in two sites per animal when the tumours were clearly palpable. Surface tumour size was measured over time. Because some tumours became bloody in some animals of the control group (empty vectors), the experiment was terminated at day 14. Data gives the mean ± SEM of the indicated group (n = 10). **A)** Animal groups injected with 100 μg pTH DNA, or 100 μg pTH-16E7SH DNA, or 50 μg plant-produced ZERA-16E7SH protein or ZERA-16E7SH protein plus adjuvant (IFA). **B)** Animal groups injected with control DNA (pTH) or plant-produced ZERA-eGFP protein.

Tumour regression in mice is associated with stimulation of cytotoxic T lymphocytes. To determine the therapeutic potential of plant-produced ZERA-16E7SH protein, the ability of ZERA-16E7SH protein to cause tumour regression in tumour-presenting mice was compared to that in mice inoculated with the DNA vaccine equivalent, pTH-16E7SH, which was previously shown to cause tumour regression [11] (Figure 3A). pTH DNA (empty vector) was used as a negative control. The ZERA-16E7SH plant-produced protein and the DNA vaccine (pTH-16E7SH) both caused significant regression of C3 tumours to a similar extent 14 days after immunization (pTH-16E7SH: 44+/-20 mm^2^; ZERA-16E7SH: 41+/-10 mm^2^), when compared to pTH which did not cause any regression.

We further tested whether an adjuvant co-inoculated with the plant-produced ZERA-16E7SH vaccine affected the cellular immune response and consequently influenced tumour size reduction. However, the addition of Freund´s incomplete adjuvant did not improve the immunogenicity of ZERA-16E7SH significantly, as further tumour size reduction was not detected (ZERA-16E7SH + IFA: 48+/-17 mm^2^; Figure 3A). This suggests that Zera® protein has an adjuvanting effect by itself, which cannot be improved under the conditions used in this study.

To determine whether the Zera® protein is immunogenic and can induce tumour regression on its own, mice were inoculated with plant-produced ZERA-eGFP protein which lacks the immunogenic 16E7 protein: the results were compared to those of mice inoculated with the empty DNA vaccine vector control (pTH). No regression of tumours was observed, with tumours growing at the same rate as those on mice inoculated with the control (Figure 3B) indicating that the immune response induced by the Zera® peptide, if there is any, does not affect tumour growth.

**Figure 4:**
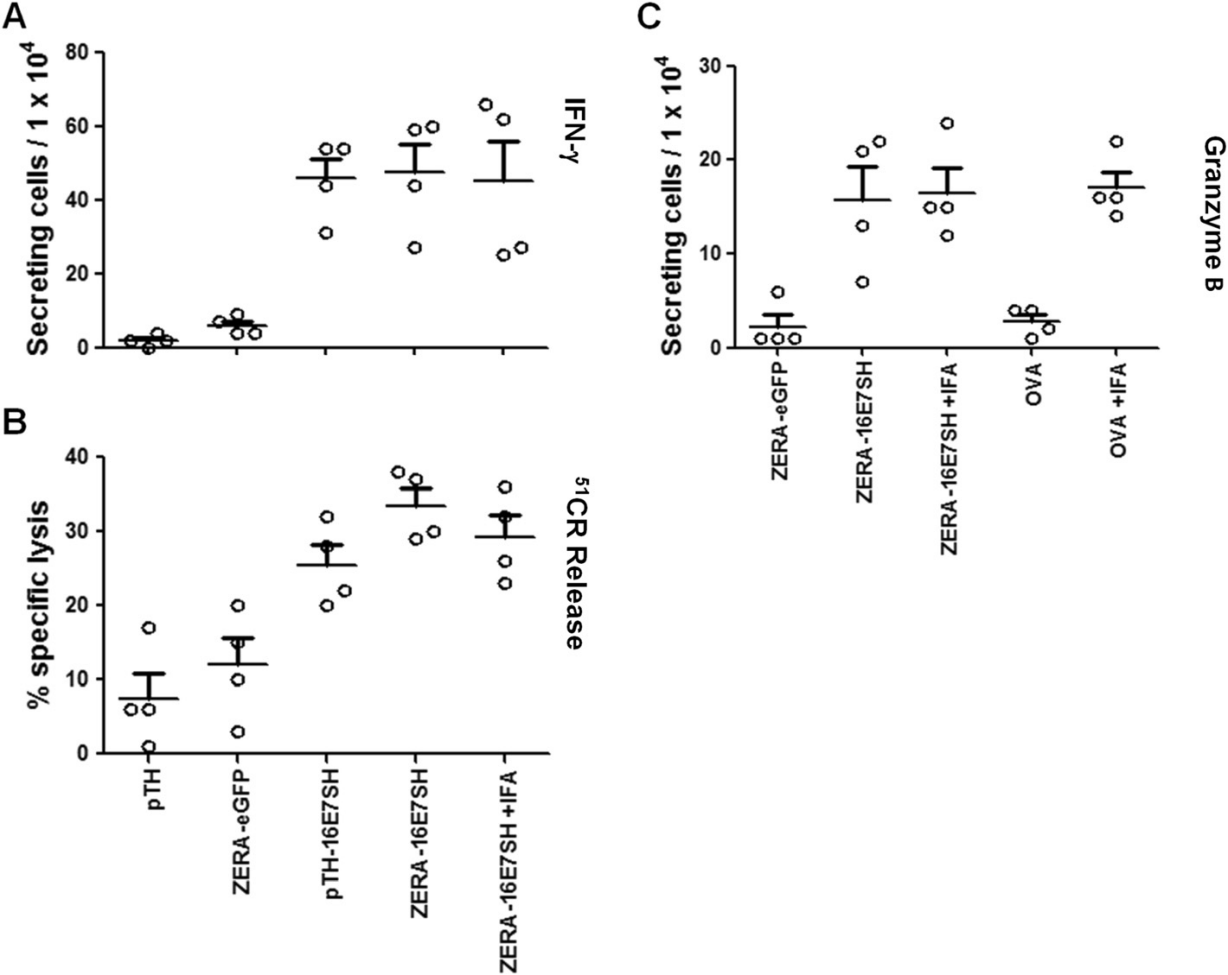
**IFN-γ response, CTL activity and Granzyme B ELISPOT assays on mouse splenocytes.** Four mice per group were injected either with 5 μg protein or with 100 μg DNA per animal, respectively. **(A)***Ex vivo* IFN-γ ELISPOT responses. Given are the means of IFN-γ secreting cells/10^4^ splenocytes ± SEM. ZERA-16E7SH compared to ZERA-eGFP (p < 0.001) and to pTH (p < 0.001). **(B)** Splenocytes were tested by ^51^Cr-release assays after one round of *in vitro* re-stimulation for lysis of E7-wildtype expressing 2 F11 target cells. Data is given as mean ± SEM. ZERA-16E7SH compared to ZERA-eGFP (p < 0.001) and to pTH (p < 0.001). **(C)***Ex vivo* Granzyme B ELISPOT responses. Four mice per group were immunized with 5 μg protein injected per animal. Given are the means of Granzyme B-secreting cells/10^4^ splenocytes ± SEM. ZERA-16E7SH compared to ZERA-eGFP (p < 0.001) and to OVA (p < 0.001).

We subsequently investigated the nature of the cellular immune response in more detail by carrying out IFN-γ and Granzyme B ELISPOT assays, as well as chromium release assays on splenocytes from the vaccinated mice. The IFN-γ assay (Figure 4A) showed that there was a significantly enhanced response caused by the plant-produced protein ZERA-16E7SH in comparison to the plant-produced protein ZERA-eGFP (p = 0.000428) and the empty DNA vaccine pTH (p = 0.000182). However, there was no significant difference measured when these results were compared to those of inoculation with the pTH-16E7SH DNA vaccine control (mean +/- SEM) or ZERA-16E7SH co-inoculated with IFA adjuvant (mean +/- SEM).

Similarly, the chromium release assay (Figure 4B) showed that the plant-produced ZERA-16E7SH caused significant specific lysis in comparison to the pTH empty DNA vaccine control (p = 0.000022) and the plant-produced ZERA-eGFP protein (p = 0.00157). Results using pTH-16E7SH or ZERA-16E7SH co-inoculated with IFA adjuvant again showed similar responses to plant-produced ZERA-16E7SH with no significant difference between them, emphasising the lack of immune enhancement using IFA.

The elevated levels of IFN-γ-secreting cells in splenocytes isolated from mice inoculated with ZERA-16E7SH as well as concomitant increased cell lysis in chromium-release assays, suggest that activated cytotoxic T-lymphocytes are responsible for the control of the tumour growth. Since Granzyme B is an important marker of activated cytotoxic T-lymphocytes and a prerequisite for the lysing of tumour cells, Granzyme B secretion assays were carried out to confirm this (Figure 4C). HPV-16 E7 wild-type expressing cells were used as targets. We demonstrated a significant response generated by the plant-produced ZERA-16E7SH in comparison to the ZERA-eGFP protein (p = 0.000609). Again, the addition of the IFA adjuvant failed to improve the immune response significantly. However, when the IFA was added as an adjuvant to ovalbumin (OVA) as a control, it generated a significant response (p = 0.000377), indicating that the IFA was viable as an adjuvant in other circumstances (Figure 4C).

### Determination of the role of Zera® protein in the immune response

Results from the above experiments demonstrate that the vaccine candidate ZERA-16E7SH is able to induce a strong cellular immune response which is able to mediate control of tumour growth. Moreover, due to the fact that IFA is normally able to enhance the cellular response of an irrelevant protein but lacks this ability when used in combination with ZERA-16E7SH, the experiments suggest that the Zera® peptide may play a role in adjuvanting the vaccine candidate which could not be further enhanced by IFA.

In order to distinguish between the role of Zera® in this immunogenic response and that of IFA, it was compared to the regression of tumours in tumourigenic mice (i) inoculated with Zera® PBs or 16E7SH proteins and (ii) co-inoculated with individual Zera® PBs and 16E7SH proteins. As we were not able to express 16E7SH alone in plants to any reasonable concentration for inoculation doses, we expressed this recombinant protein in *E. coli* as this method resulted in the production of adequate amounts of 16E7SH easily. In addition, the Zera® PBs used in these experiments were produced in insect cells as their expression using this method is high (+/- 40 mg/L), and recovery is very efficient. Zera® PBs from insect cells and tobacco plants are also very similar in that they are round, range in size from 0.5 to 1 μm, membrane-surrounded and do not undergo post-translational modification [32].

**Figure 5:**
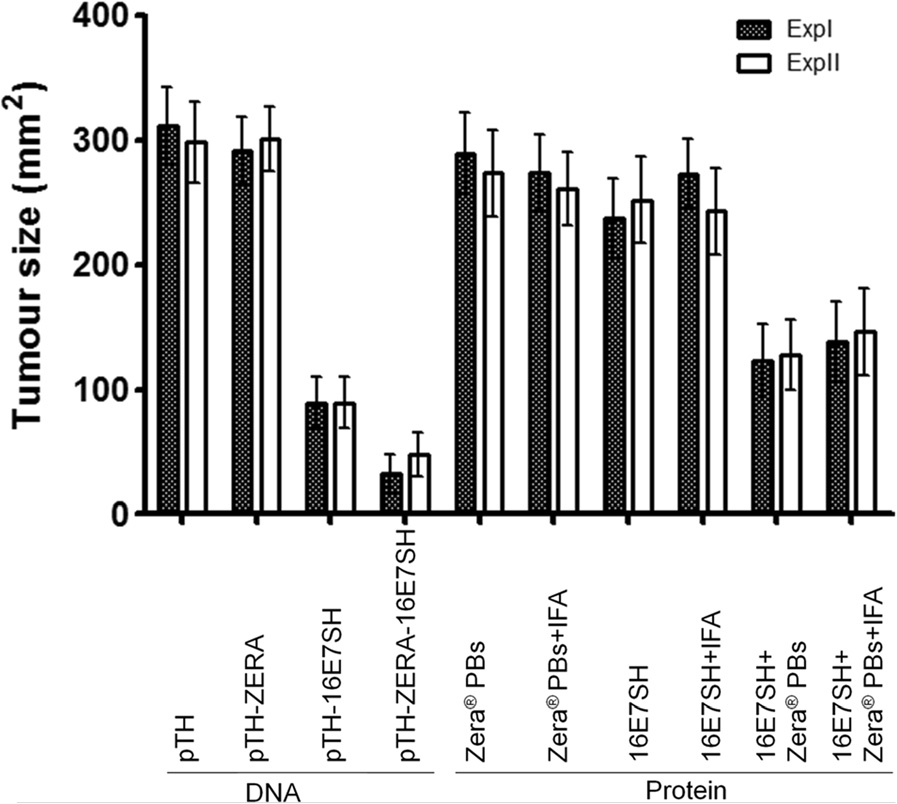
**Tumour regression in mice inoculated with Zera® separately.** Mice received 0.5 × 10^6^ C3 tumour cells s.c. and when the tumours were clearly palpable they were immunized with 100 μg DNA vaccine or 2.5 μg 16E7SH +/- 2.5 μg Zera® PBs +/- 100 μl IFA per animal and surface tumour size was measured. Experiments were repeated (Exp. I and Exp. II) and both results are shown. The experiment was terminated at day 39 due to the size of tumours in the control groups (empty vectors). Data gives the mean ± SEM. of the indicated group at day 39 but in the second exp: on day 43 (n = 10). pTH-ZERA-16E7SH DNA compared to pTH-16E7SH (Exp. I - p < 0.05, Exp. II p = 0.2). 16E7SH protein compared to 16E7SH protein plus Zera® PBs (Exp. I - p < 0.005, Exp. II p < 0.0005).

This experiment was repeated twice and the results presented in Figure 5 show that only mice co-inoculated with the 16E7SH and Zera® proteins showed a significant tumour regression. 16E7SH protein did not cause any significant reduction in tumour size. Similarly, Zera® protein alone did not cause tumour regression, indicating that Zera® has no anti-tumour properties on its own. Additionally, as observed previously, the inoculation with IFA did not modify the tumour regression efficacy of any of the inoculations tested.

Inoculation of mice with the cognate DNA vaccines (pTH-16E7SH and pTH-ZERA-16E7SH) also confirmed their ability to cause tumour regression, with pTH-ZERA-16E7SH showing a significantly stronger control of tumour growth. The corresponding inoculation with pTH-ZERA also did not have any effect in decreasing tumour growth (Figure 5). With these experiments we could clearly demonstrate that Zera® has an immunostimulatory role which cannot be substituted by an adjuvant.

Splenocytes from these mice were also subjected to IFN-γ and Granzyme B ELISPOT assays, as well as chromium release assays to determine the nature of the immune response in the mice.

### IFN-γ secretion of cytotoxic T lymphocytes

**Figure 6:**
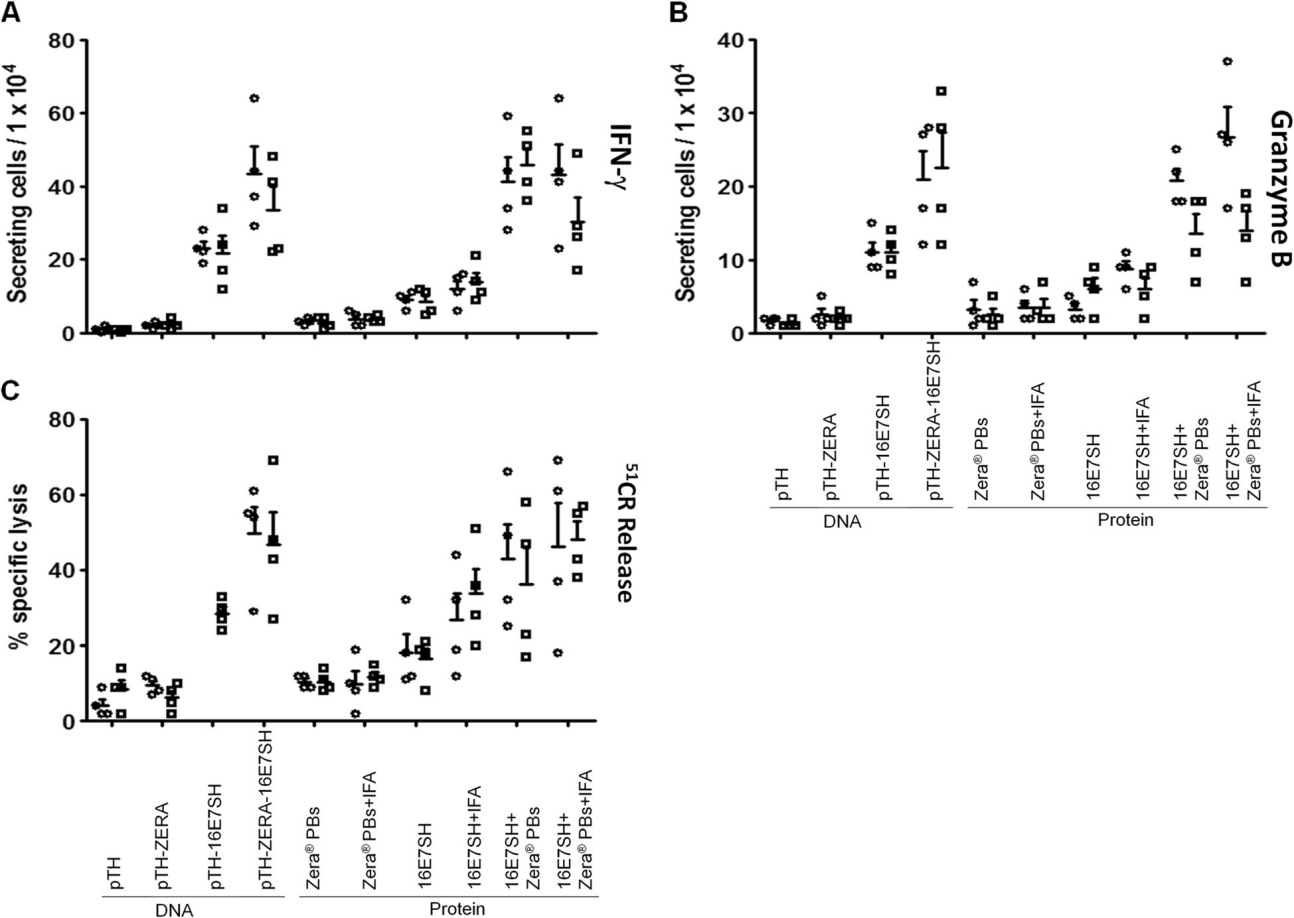
***Ex vivo *IFN-γ, CTL activity and Granzyme B ELISPOT responses in mice inoculated with Zera® separately.** Two groups of four mice were immunized with 100 μg DNA vaccine, or 2.5 μg 16E7SH +/- 2.5 μg Zera® PBs +/- 100 μl IFA per animal for each assay. **(A)***Ex vivo* IFN-γ ELISPOT responses. Given are the means of IFN-γ secreting cells/10^4^ splenocytes ± SEM. **(B)***Ex vivo* Granzyme B ELISPOT responses. Four mice per group were immunized with 5 μg protein injected per animal. Given are the means of Granzyme B-secreting cells / 10^4^ splenocytes ± SEM. 16E7SH protein compared to 16E7SH protein plus Zera® PBs (Exp. I - p = 0.0001, Exp. II p < 0.05). **(C)** The splenocytes were tested by ^51^Cr-release assays after one round of *in vitro* restimulation for lysis of syngeneic E7-wildtype expressing 2 F11 target cells. Data gives the mean ± SEM of the indicated group (n = 4). One representative of the two experiments is shown.

IFN-γ levels of splenocytes isolated from mice inoculated with DNA in 2 biological repeat experiments showed elevated amounts in mice inoculated with pTH-16E7SH and pTH-ZERA-16E7SH DNA, compared to those inoculated with pTH-ZERA or pTH DNA only (Figure 6A). The Zera®-containing construct led to a stronger induction of IFN-γ secretion: this was statistically significant (p: 0.04) in Exp. I and not (p: 0.2) in Exp. II. IFN- γ assays on sera from mice inoculated with 16E7SH protein alone showed only very moderate responses which were not significantly enhanced by IFA. This outcome corresponds with data shown earlier (Figure 5). Importantly, the IFA lot used in this study enhanced the immunogenicity of a protein-based vaccine in another context (data not shown), proving that its lack of boosting the immune response was not due to malfunction of the adjuvant.

Interestingly, a comparison of the mice inoculated with 16E7SH protein with those co-inoculated with 16E7SH protein and Zera® protein (PBs) shows a clear enhancement of the immunogenicity by Zera® PBs, with both experiments showing statistical significance (p: 0.004 in Exp. I and 0.0003 in Exp. II). When IFA adjuvant was added to the inoculation dose consisting of 16E7SH and 16E7SH and Zera® PBs, the responses of animals were similar, again showing that Zera® PBs enhanced the immune response.

### Granzyme B ELISPOT assays

The Granzyme B ELISPOT assays performed on samples from mice inoculated with DNA showed similar trends (Figure 6B). Comparison of samples pTH-16E7SH and pTH-ZERA-16E7SH indicate an elevated immune response compared to pTH-ZERA or pTH alone, although these responses were not statistically significant for either of the biologically repeated experiments (p: 0.06 in Exp. I, p: 0.06 in Exp. II). The Granyzme B ELISPOT assays performed on samples from mice inoculated with protein showed a significant difference when comparing the effect of 16E7SH protein alone and the effect of 16E7SH protein and Zera® PBs (p: 0.0001 in Exp. I, and 0.03 in Exp. II). The effect of adding IFA to 16E7SH and to 16E7SH + Zera® PBs did not show a significant increase in Granzyme B-secreting cells, confirming that Zera® has an adjuvanting effect.

### Chromium release assay

Chromium release assays carried out on splenocytes from vaccinated mice showed a trend indicating an enhanced response (lysis of target cells) (Figure 6C). This included the comparison of the response between mice inoculated with pTH-16E7SH and those inoculated with pTH-ZERA-16E7SH (p: 0.0849), which was similar to the observations between cognate samples measured in IFN-γ and Granzyme B secretion assays (Figure 6A and B). A trend of increased cell lysis was observed when comparing the response of mice inoculated with 16E7SH protein to those co-inoculated with 16E7SH protein and Zera® PBs, although the measurements were not statistically significant (p: 0.0513 in Exp. I and p: 0.1051 in Exp. II) (Figure 6C). A similar trend was observed in the IFA-supplemented groups (16E7SH protein + IFA and 16E7SH protein + Zera® PBs + IFA), where again IFA did not enhance immune responses.

### Humoral antibody response

**Figure 7:**
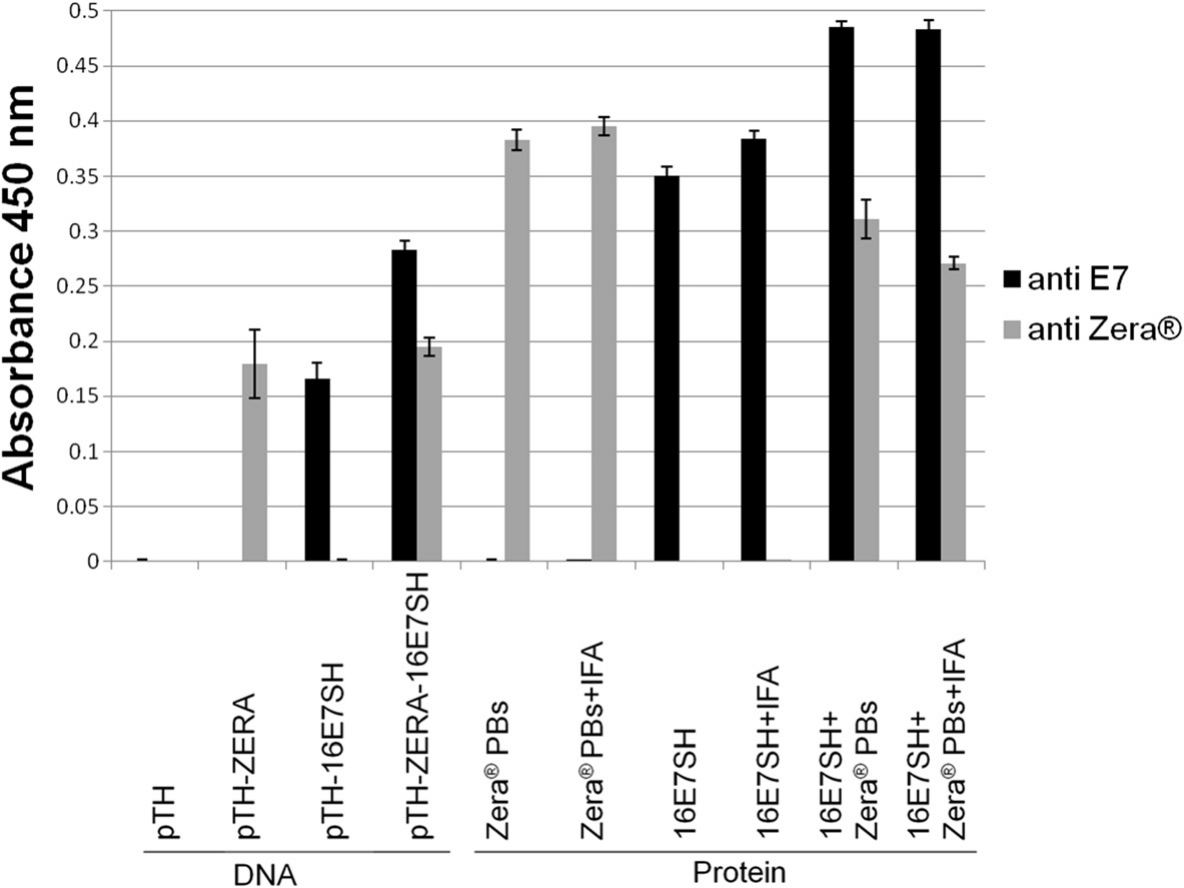
**Humoral response of mice inoculated with Zera® separately.** Four mice per group were immunized with 100 μg DNA vaccine or 50 μg protein and blood was taken post-immunization (day of splenectomy). Direct ELISA was performed against both Zera® and 16E7 protein using anti-Zera and anti-16E7 IgGs. Data gives the mean ± SEM of duplicates of the indicated group (n = 4). One representative of the two experiments is shown. pTH-ZERA-16E7SH DNA compared to pTH-16E7SH (p < 0.005), 16E7SH protein compared to 16E7SH protein plus Zera® PBs (p < 0.005).

We further wanted to determine if Zera® generates an improved humoral immune response after DNA and protein vaccination. We therefore investigated the presence of anti- Zera® and anti-16E7 IgG in serum samples from vaccinated mice using an ELISA. The comparison of the pTH-16E7SH- and pTH-ZERA-16E7SH-treated groups showed the induction of significantly increased levels of anti-E7 IgG by the ZERA-construct (p: ≤0.0022) (Figure 7). As expected, anti-ZERA IgG was only detected in the pTH-ZERA and pTH-ZERA-16E7SH-treated group. The 16E7SH protein induced an anti-E7 IgG response that was significantly enhanced when co-inoculated with Zera® PBs (16E7SH + Zera® PBs) (p: ≤0.0025) (Figure 7). When mice were inoculated with Zera® PBs alone, they were able to induce high levels of anti- Zera® IgGs as expected, but there was no enhancement detected in mice inoculated with Zera® PBs + IFA (Figure 7). Interestingly, again IFA did not cause an enhanced anti-E7 IgG response when comparing samples from mice co-inoculated with 16E7SH protein + Zera® PBs with or without IFA.

## Discussion

There is a need for therapeutic HPV vaccines that eliminate existing lesions and malignant tumours by inducing cell-mediated immune responses against HPV-infected cells. Presently, various strategies such as DNA based, peptide- and protein based and live-vector based vaccines are utilized [12]. DNA vaccines have emerged as potentially useful HPV therapeutic vaccines, but there is limited success after clinical trials of DNA vaccines so far and in the actual commercialization of the product. Moreover, the fear of DNA integration into the host genome and subsequent genomic instability remains. Therapeutic protein vaccines, on the other hand, are generally considered safe. One drawback of protein vaccines is that they are often not very immunogenic, and often need either to be fused to other immunogenic proteins, or have adjuvant added, in order to increase their immunogenicity. Production of protein vaccines can also be very costly when it is done in mammalian cells and the concern of contamination by other human or human-infecting viruses remains. Plant-produced proteins could provide an alternative as they can be produced economically and have been shown to be safe for use in humans [16].

In this study we investigated if the plant-produced shuffled HPV16 E7 protein fused to Zera® is a suitable candidate as a therapeutic vaccine for HPV-16 infections and HPV-related tumours. An artificial shuffled HPV-16 E7 gene (16E7SH) was selected as it has previously been shown to cause regression of tumours in mice when tested as a DNA vaccine. 16E7SH was fused to Zera®, a novel signal sequence which promotes the formation of protein bodies during expression of fusion proteins. We wanted to investigate the role of Zera® in increasing 16E7SH production in plants, and also its role in enhancing the immunogenicity of the plant-produced protein. Lastly, the possibility that free Zera® could act as an adjuvant was explored by mixing Zera® PBs with the purified 16E7SH protein.

For safety reasons the use of wildtype HPV-16 E7 for vaccination is not feasible in humans. Approaches like the introduction of point mutations into the E7WT gene, however, lead to an unwanted loss of naturally occurring epitopes that is potentially associated with a decrease in vaccine efficacy. We used a rearranged (“shuffled”) E7 sequence which lacks transforming properties [11]. Ultimately this non-transforming HPV-16E7SH supplies all potential naturally-occurring T cell epitopes, covering the broad range of MHC restriction. Consequently, prior knowledge of the patient’s HLA-haplotype is not required which is especially important in the outbred human population. In addition, a more potent immune response may be induced, involving all occurring HLA-restriction elements in the vaccine.

We tested whether 3 different recombinant HPV16 E7-derived proteins could be produced in plants. The expression in plants of recombinant protein from constructs encoding 16E7SH alone, 16E7SH fused to Zera®, and Zera® fused to wildtype HPV-16E7, was assessed by comparing expression at 3, 5, 7 and 10 dpi. ZERA-16E7SH and ZERA-16E7 proteins were successfully expressed in plants as 29 and 24 kDa fusion proteins, respectively. However, the expression of 16E7SH alone was not detected, indicating that fusion with Zera® enhanced accumulation levels of the protein in plants. The incorporation of a silencing suppressor increased ZERA-16E7SH accumulation by 6-fold, indicating that post-transcriptional gene silencing (PTGS) plays a role in the expression of the proteins.

This increase with addition of a silencing suppressor is lower than other reported increases, such as the 30-fold increase measured using with GFP expression [33]. However, the accumulation of ZERA-eGFP reached ±25 g/kg in our experiments (data not shown), which was more than 70-fold higher than the GFP expression reported by Voinnet et al. [33].

The Zera®-HPV proteins were expressed at levels ranging from 0.1 - 6 g/kg. ZERA-16E7SH levels were the highest, varying from 1 - 6 g/kg. This was 2-fold higher than that obtained by Massa et al. [20], who attained levels of 0.4 g/kg for E7 fusion protein production in *N. benthamiana.* In contrast, expression of the 16E7SH protein alone was too low to quantitate, due either to a very poor level of expression, and/or to degradation during extraction.

The time trial expression profiles showed stable protein accumulation up to 12 dpi in samples infiltrated with ZERA-eGFP and for the ZERA-16E7 constructs. This is much longer than seen in previous studies where the expression of the proteins peaked at 60-70 h, even with the addition of a silencing suppressor [33]. Protein degradation in the cytoplasm is one of the reasons behind this decreasing protein concentration, which is alleviated by the sequestration of the proteins into PBs. As the Zera® fusion protein is translated, it is directly sequestered into ER-derived and membrane-delimited protein bodies. It is thought that this encapsulation protects the fusion protein from proteolytic degradation. At the same time, the PBs serve as a very useful means for purification of the proteins, as they are dense organelles which can be easily purified on density gradients [26].

The ability of the plant-produced ZERA-16E7SH PBs to cause tumour regression was shown to be significant and similar to that of the DNA vaccine equivalent. Further co-inoculation of ZERA-16E7SH PBs with adjuvant, however, did not significantly enhance tumour regression suggesting that Zera® has an adjuvanting effect by itself. Inoculation of tumourigenic mice with plant-produced ZERA-eGFP lacking 16E7SH also did not result in tumour regression suggesting that Zera is not immunogenic. Despite the fact that it has been shown that eGFP is minimally immunogenic in C57/BL6 mice [34], the lack of tumour regression observed when mice were inoculated with ZERA-eGFP further supports the evidence that the 16E7SH is the immunogen causing tumour regression and that Zera® does not contribute to this.

The pTH-ZERA-16E7SH construct appeared able to induce higher immune responses than the Zera®-free counterpart (pTH-16E7SH); however, the data were not in all cases statistically significant. In general, the DNA-based vaccines were more efficacious than the protein-based vaccines in the context of control of tumour growth (Figure 5), which probably reflects in part the fact that DNA vaccines induce more Th1 than Th2 responses after intramuscular injections. The pTH-16E7SH construct did, however, also induce a moderate IgG response, as measured by ELISA (Figure 7). Additionally, IFN-γ levels of splenocytes isolated from mice inoculated with pTH-ZERA-E7SH were elevated compared to mice inoculated with pTH-16E7SH (Figure 6). This trend was also observed in Granzyme B ELISPOT assays as well as in chromium release assays.

One way to enhance potencies of DNA vaccines is to increase antigen expression in professional antigen presenting cells, such as dendritic cells. As ZERA-16E7SH is expressed well in plants, and 16E7SH is not, it can be speculated that ZERA-16E7SH is also expressed to much higher levels from a DNA vaccine in mammalian cells than 16E7SH alone, and that this would in turn enhance CTL response induced by this DNA vaccine [12]. In fact, the increase in protein accumulation due to fusion with Zera® and production of PBs has been observed in mammalian cells as well as other organisms [32]. It can be speculated that PBs are also formed in the mammalian cells that are inoculated with the DNA vaccine. It remains unclear how such heterologous organelles, which typically are retained in the ER, could in turn enhance the immune response as we observed in this study.

In our study we showed that Zera®-PBs were clearly able to stimulate humoral and cellular immune responses either as ZERA-16E7SH fusion protein or as Zera® PBs co-inoculated with 16E7SH proteins. Normally, subunit protein vaccines are not very efficient in the induction of the cellular immune responses; thus, this finding could be of great interest in the context of protein-based therapeutic vaccines. It is well known that professional antigen presenting cells like DCs and macrophages favour the uptake of particles with repeating sequence motifs. The adjuvanting effect of Zera® could also be due to its particulate nature. Interestingly, in our hands IFA was in no case able to enhance immune responses significantly in the presence of Zera®, which was wholly unexpected. The same IFA lot used in the present study induced an enhancement of the antibody response after immunization with other antigens (highly purified recombinant OVA (data not shown)). In addition, despite the fact that IFA is commonly used to enhance Th2-directed responses, we observed in the OVA immunizations an effect on the Th1 response.

## Conclusions

In summary, we were able to transiently express a ZERA-16E7SH fusion protein to very high levels in plants, in contrast to the free 16E7SH protein. We were able to demonstrate that fusion with the Zera® peptide significantly enhances expression of a HPV 16E7SH protein in plants. The Zera® fusion protein and Zera® PBs mixed with the 16E7SH protein alone enhanced cellular and humoral immune responses to 16E7SH. These responses could not be further enhanced by the addition of a common adjuvant, and we speculate that Zera® has adjuvanting capabilities and is able to enhance CTL and antibody responses in both DNA and protein vaccines. We feel we have demonstrated proof of efficacy in a mouse tumour model of a novel HPV therapeutic vaccine candidate, which should be easy and cheap to produce and purify. For further development of this therapeutic vaccine, a DNA prime followed by protein boost might be ideal in order to achieve further enhancements in immunogenicity.

## Abbreviations

16E7SH: Artificial shuffled HPV 16E7; PB: Protein body; PTGS: Post-transcription gene silencing; IFA: Incomplete Freund’s adjuvant.

## Competing interests

This work was supported by a contract between ERA Biotech SA and the Department of Molecular and Cell Biology, University of Cape Town. Part of the results of this work is subject matter of the patent application WO2010040847.

## Authors’ contributions

MW created the expression constructs, carried out transient expression experiments, helped with animal experiments, did serum analysis and drafted the manuscript. PÖ supplied the 16E7SH, carried out animal experiments, analysed data from animal experiments and helped in drafting the manuscript. FNA participated in the mouse experiments; PM helped in design of the experiments and in purification of the *E. coli* produced proteins; LA expressed and purified PBs; AEM participated in design of the study and helped to draft the manuscript; IIH conceived, designed and coordinated the study, helped drafting the manuscript and revised the paper. EPR participated in the design of study and drafting of manuscript. All authors read and approved of the final manuscript.

## Pre-publication history

The pre-publication history for this paper can be accessed here:

http://www.biomedcentral.com/1471-2407/14/367/prepub
